# Extracts

**Published:** 1881-11

**Authors:** 


### Extracts.

CATALEPSY.—You will recognize it as a nervous or hypnotic condition, unattended by danger, by several facts, viz: there is no fever, no acceleration of the pulse, the respirations are normal, and if the patient be madé to take food the vegetative functions will go on as usual. It may be that such a case might be successfully treated with the cold douche, but we do not see cases of catalepsy sufficiently often to test any plan of treatment thoroughly. In the case of prolonged somnolence to which I have just referred, the patient was finally brought out of it by firing. A common hammer, for instance, sufficiently heated, may be applied over the spine. It has not yet been tried in this case, for I wished to give you an opportunity to see one such case as it will probably be the only one you will ever see.

LEAD COLIC.—Here is a patient who is suffering, and has suffered for several days, from abdominal pain. He has no fever; the pulse is somewhat diminished in frequency, apparently; there is no tenderness on pressure over the abdomen; on the contrary, firm pressure with the open palm affords a certain degree of relief. You see the abdomen is not tympanitic, is not distended. He has constant pain here, increased in exacerbations.

Now, gentlemen, with this group of symptoms, what would you look for? We have no evidence of inflammation. If, then, we exclude inflammation, and the pain be the most prominent symptom, it is a neuralgic affection. This is properly a neuralgic affection, although it goes by the name of colic. The term colic really implies a condition which we suppose to be a spasm. Now, in this patient we have an example of what is called saturnina, or lead colic, an affection which has a variety of names. How do we know it? In the first place the man's occupation would suggest it; he is a painter, and is exposed to lead. But we have still more positive proof in a blue coloration along the gums, formed by a combination of lead with sulphuretted hydrogen, and it is quite well marked here.

This patient was treated by sulphate of magnesia and dilute sulphuric acid, but on account of the irritability of the stomach it was not retained. He is now taking iodide of potassium. The iodide of potassium has been used for many years in cases of lead poisoning, on the theory that it combines with the lead in the tissues of the system, forms a soluble compound which is eliminated through the kidneys. I must confess some skepticism in regard to this, but as there is no other remedy which may be considered as having any particular effect, this is, at present, the one in general use. This patient will recover; it is only a question of time. It has been heretofore very much the practice to purge patients. I do not see any good object to be attained by it; I doubt its utility. It seems to me all that is required in this line is to relieve the constipation.—*Clin. Lecture, by Austin Flint, M.D., in Nashville Journal of Med. and Surg.*

---

A Case of Hysteria.—I saw the patient of a Monday. She was in bed, and so unconscious that only by repeated efforts could partial protrusion of the tongue be obtained. Her extremities were in a constant and irregular motion, legs and arms were jerking simultaneously and alternately. I was told that these convulsive movements had lasted without intermission, except during sleep, for six weeks.

Further examination revealed a loaded tongue, foul breath, and a tumid abdomen. The bowels had not moved since the preceding Friday.

I left a good cathartic dose, of which podophyllin was an ingredient, and began the moral treatment by some strong remarks to her mother on the certain curability of the case, but that it might require severe measures. I had but little faith in her unconsciousness.

The next day she was better, so as to put out her tongue and answer in monosyllables; the convulsive movements the same. I left another cathartic and gave another dose of "talk!" "It was a pity to see a fine girl suffer so! She could be cured, but sometimes we had even to burn the spine with a hot iron to save such cases!" The next day this was repeated and a tonic prescribed, as the condition of her digestive organs was much better. On the fourth day, the convulsive movements continuing, I proceeded to ulterior measures. I assured the mother that I could cure her, and obtained her assent to do as I pleased, promising that I would not injure her daughter. A strong neighbor woman was brought in and instructed as to what I wanted her to do. I provided a large pitcher and a pailful of cold water from the well and a washtub. The patient was drawn across the bed, her head and shoulders over the tub, the assistants holding a shoulder and an arm on each side. Then, on her upturned face, I poured the water in a steady stream, and from as great a height as I could raise my arm. She struggled, tussled and fought, but was held firm. I had a good hold of her back hair with my left hand. She opened her mouth to scream, but the stream of water was directed into it, and she tried that but once more. I did not draw on the pail of water. Before the ewer was empty the convulsions had ceased! They have not returned since! As I assisted in drying her, with many expressions of sympathy and shame for treating a young woman in such a manner, they gave indications of beginning again; but a movement toward repeating the douche promptly checked them. She is not yet well, but she has had no more convulsions.—*J. C. Reeve, M.D., in Ohio Medical Journal.*

Would not a few inhalations of chloroform, cautiously administered, accompanied, if necessary, by an anodyne, have relieved the symptoms without resort to violent means of treatment? Hysterical patients say they *cannot;* to an observer it often appears as if they *will not*; the truth is they *cannot will.* This important fact should be borne in mind in adopting any method of treatment.

---

Diphtheria.—It occurs sporadically by preference in the winter months, and, while the young are especially its victims, it nevertheless is known to occur in extreme old age; while croup is a disease peculiar to infantile life. Again, in croup the deposition of the membrane is confined to the throat; while in diphtheria it may occur wherever there is a mucous membrane; and not only in these situations, but wherever the cuticle layer of the skin has been abraded, a diphtheritic patch may make its appearance. The membrane once expelled in croup, the disease no longer exists; in diphtheria your visits to the patient may have ceased, when all at once the disease is lighted up again, and perhaps with renewed severity. Again, it is not unusual to find albuminuria in diphtheria, but very rarely is this manifestation present in croup.

One result among the sequelæ is to my mind pathognomonic as to the nature of the pre-existing disease; that is, paralysis; the involvement of the deeper structures which occurs interferes with the function of the nerves distributed to the parts. Uusually the soft palate will hang flaccid for weeks or months. Nothing is more common than to have muscular weakness, if not paralysis, following an attack; a frequent seat of this muscular affection being found in the ocular muscles.

Now, as to treatment. If these points which I have brought forward be true, then nothing seems more conclusive than that our treatment should be both local and constitutional. The constitution, as I have said, is usually invaded before you are called to see the case; and, since we regard it as a blood dyscrasia, that constitutional treatment should be directed to supporting the life of the blood-corpuscles. You attempt to secure that object in erysipelas and hospital gangrene; and this, as I have said before, is to be regarded as a disease of that type. A disease fraught with danger from the start, you may well imagine, requires not only accurate diagnosis, but prompt and decisive measures of treatment. Muriated tincture of iron, freely and persistently given, should form the basis of your treatment; its astringent property tends to prevent the organization of the membrane, while its effects upon the constitution tend to sustain the life of the patient, and thus enable him to throw off the membrane.

Another point I wish to speak of is this: the use of alcohol has been brought forward as something new by members of the medical profession. Any poison in the blood tending to destroy life by destruction of the blood-corpuscles, as well as by its debilitating effects upon local tissues, indicates a stimulant and a preservative. In alcohol we have the most powerful agent to prevent the destruction of animal tissues. Its stimulant property needs no comments. Give muriated tincture of iron, then, and alcohol with nourishment, but give the iron according to Col. Mulberry Seller's directions for the use of his eye-water [“internally, externally and eternally”]. Give along with this a whisky toddy, a milk punch, or, what is better, Christmas egg-nog, and don't withhold it out of fear of intoxication. The amount of alcohol that can be taken under these circumstances with immunity is astonishing. I have myself given a child, seven years old, a pint of whisky in twenty-four hours, without producing any symptom of intoxication. Now, these, gentlemen, are fundamental principles. Do not, I beg you, in such a case go off into speculations as to the value of carbolites. Carbolic acid is fit for nothing but to smell, and even for that purpose it may be very handsomely substituted. Neither is it necessary to give the sulphites, nor the hyposulphites, nor chlorate of potassium. We have recently seen from Dr. Jacobi, of New York, what we might have supposed before—that is, that in excessive doses chlorate of potassium is a virulent poison. Then let me insist upon your strict adherence to muriated tincture of iron. Make it a religious duty, and your experience, if it correspond to mine, will give you cause for congratulation.—*John A. Larrabee, M.D., in Philadelphia Med. Times.*

---

Is Gonorrhœa Specific?—Dr. P. Albert Morrow (*New York Medical Journal and Obstetrical Review*) discusses the question of the specific or non-specific nature of gonorrhœa. After a close analysis of the arguments for and against specificity, he concludes that the latter are pure hypothesis, and wholly untenable, while all the facts experimental, clinical, and pathological, are in faver of the non-specific character of gonorrhœal inflammation. He claims that when the gauge of specificity is applied to gonorrhœa it corresponds to none of the conditions of an undoubtedly specific inflammation. No artificial production of any disease belonging to this group is possible; a specific disease is the product alone of a specific poison. Gonorrhœa, on the contrary, may be due to a variety of causes, contagious, irritant, mechanical or chemical, diathetic, etc. Again, in all specific diseases there is applied a period of incubation. No incubation, properly so called, characterizes gonorrhœa. A drop of this same gonorrhœal pus, which may require two or three days to excite suppuration of the urethra, will develop such effects in a few hours when applied to the conjunctiva, showing that the so-called incubation depends not upon the quality of the exciting cause, but upon the susceptibility of the mucous membrane. Another distinctive peculiarity of this group is that a single attack of the disease confers almost complete security from another attack, a peculiarity precisely the opposite of what is observed of gonorrhœa. The morbid poison of a specific inflammation, once in action, continues until the textural predisposition to its special stimulus is exhausted. The patient is incapable of regenerating the poison or of being affected by it when exposed anew. Both of these conditions are negatived in the clinical history of gonorrhœa. Finally, specific inflammation determines special pathological changes and demands special treatment. Identical pathological processes are met with in urethritis from various causes, and the most radical of virulists treat all urethral inflammations alike. Gonorrhœa, arising from other causes than contagion, is widely different from that so produced, and the variability in the time of incubation is in common with many other indisputably specific diseases. The argument in relation to the action of gonorrhœal pus on the conjuctiva is of no value; the same thing has been noticed as to the effect of diphtheritic exudation. While Dr. Morrow's argument seems strong, the same logic would deny specificity to diphtheria. The weakest part of his argument is that based on pathology.—*Chicago Med. Review.*

---

GONORRHŒAL RHEUMATISM.—(Clinic of Prof. J. M. Da Costa.) Gentlemen: At the conclusion of my last lecture I had just presented, and was about making some remarks upon, this case, which you will recall as that of the young man who had suffered with inflammation of the left ankle and knee joint, in whom the trouble arose in connection with, or, if you like, in consequence of, co-existing gonorrhœa. I will not go over the points I have already enumerated, but shall merely say, there still exists a good deal of tumefaction of the joints, and that being fully convinced of the existence of pus in the left knee joint, we aspirated the swelling, with the result of obtaining a considerable amount of pus, which made the case a clear one, and a typical illustration of the form of disorder known as gonorrhœal rheumatism. Suppurative synovitis, then, is characteristic of this form of joint affection. He did very well for the first few days after admission, under the treatment proper for ordinary rheumatism, when his urethral discharge was as yet unknown to us. We soon began to observe, however, his peculiar fluctuating temperature, which ascended each afternoon and fell in the morning. The urethral discharge was next discovered, and soon after decided fluctuation appeared in the knee, and, by aspiration, more than eight ounces of pus were obtained, the most of which seemed to come from around the joint rather than in it. After this operation he felt better, but in a few days the temperature went up again although the knee exhibited no fresh accumulation of pus and, indeed, was still discharging, though less freely. We then detected obscure fluctuation about the right shoulder, deeply situated. Having the history of the case before us, we, of course, had no difficulty in determining what this meant; the fluctuation around the shoulder could have but the same interpretation as that of the knee joint, and we did for it exactly what we did for the knee, with the exception that in place of aspiration we made an incision at the lower point of fluctuation, near the angle of the scapula, and as much as twelve ounces of pus were obtained. Let us look at this point. Here you see the incision, below the line of the scapula, from which pus is discharging. There is no swelling in the joint proper; the whole amount of pus seems to have been in the deeper tissues immediately around the joint; in the knee, you remember, we had some occasion for believing that there was pus in the joint as well as around it. This is a good illustration of what occurs in gonorrhœal rheumatism, pus in and around the joints. All the time while this is going on he has a fever; the temperature is more or less high, with slight remissions, and the rheumatic symptoms are prominent, and yet there are no heart symptoms whatever. You may recall that, in regard to his treatment, we had placed him upon chlorate of potassium, with a view of modifying the state of the diseased mucous membrane, and, to some extent, the blood; and, as I told you in our last clinic, a certain amount of chlorate of potassium (gr. v solution) was employed locally, as a urethral injection. No other local treatment was adopted. This treatment shall be continued until the gonorrhœa is entirely stopped, when we shall suspend the chlorate of potassium, and give tinct. ferri chloridi (gtt. xx) four times daily, ten grains of quinia daily, and good food. He is now getting well; there are no fresh depots of pus forming, and I do not think that they will form, as he is entering into convalescence. I only say this from present indications, however, as his fever has nearly gone and no new joints are involved. This case demonstrates what I am in the habit of teaching, that gonorrhœal rheumatism is, in truth, a form of pyæmic rheumatism, involving special features and requiring special treatment. The much less frequent occurrence of acute heart complications in this disease serves also to separate it clinically from ordinary acute articular rheumatism.—*College and Clinical Record, July* 15, 1881.—*American Specialist, September*, 1881.

---

Venesection.—T. M. Dolan, F.R.C.S.Ed., L.R.C.P.Ed., (*Medical Press and Circular*) in a paper upon the neglect of local and general blood-letting, says that the following propositions may be maintained:

1. Venesection has no direct influence over inflammation, external or internal. 2. Venesection is useless in the case of all external inflammations. 3. Venesection is of use in those inflammations, where the cardiac and respiratory functions are interfered with. 4. Local bleeding in external inflammations is most useful, its effect is patent. 5. Local bleeding in internal inflammation, where there is a direct capillary circulation between the skin and inflamed part, is of manifest service. 6. The benefit of local bleeding, where there are not such conditions, is neither clear nor positively ascertained.

He sums up and closes his paper in the following words of Dr. Markham:

"Is it credible that a remedy which, through evil report and through good report, has steadily held its own in the catalogue of curative agencies, from the days of Hippocrates to our own, can all at once have ceased to be of service to humanity? Must we believe that all the great minds, who, through the long ages of past medicine, have resorted to this remedy, have been using it under a delusion? Surely, the very fact of the antiquity of the remedy, its universality, and its persistence during all times as a curative measure is strong *a priori* evidence of its possessing value and excellence as such."—*Louisville Med. News.*

---

Alkaline Water as a Vehicle for the Iodides and Bromides.—E. C. Seguin states that he finds it practicable to give the iodide of potassium and the various alkaline bromides without risk of disturbing the digestion by the following method: His plan includes three almost equally important conditions:

(1) The use of a simple aqueous solution of the salt.

(2) Its ingestion upon an empty stomach (fifteen or thirty minutes before food).

(3) Its very free dilution with an alkaline solution.

He directs his patients to measure out their dose of iodide or bromide (he always uses a simple aqueous solution of the salts), into a glass and add a liberal quantity of Vichy water, from one half to a whole glassful. In cities the siphons of artificial Vichy water can readily be procured. Where patients are so situated that they cannot obtain these he has them use the effervescent salt, a teaspoonful of which, in a glass of water, takes the place of the water from the siphon. When patients cannot afford to buy these preparations he directs them to add a good-sized pinch of bicarbonate of soda to a glass of water containing the prescribed dose of iodide or bromide.

By this method of administration the supposed irritating effect of the salts upon the mucous membrane of the stomach is reduced to a minimum, if not absolutely neutralized, and he is able to administer sixty to one hundred or more grains of the bromide in the day without any gastric disturbance, and of the iodide of potassium he has given as much as ℥j. in the day without causing indigestion. Furthermore, the sparkle and sub-acid taste of the effervescent drink mask the saline taste of the iodide and bromide very effectually.—*Archives of Med.—St. Louis Courier of Med.*

---

Food Stuffs Under the Microscope.—22. "Mellin's food." *Claim*, to be a perfect infants' food. Evidently a cooked preparation, as the wheat-starch was broken up and indifferent to polarized light. Four gluten cells, two without coat. Some emptied gluten cells. Hairs of beard of wheat. Granular masses of cooked substance. Preparation very sweet with sugar.

23. "Horlock's food." *Claim*, perfect infants' food. Tegument. Hairs. Starch and starch bundles of wheat evidently. No polarization. Hence, cooked. Considerable number of gluten cells.

24. "Imperial granum." Specimen obtained from consumer in New York City. *Claim*, "very rich in phosphates and gluten cells." A very careful morphological examination disclosed only *starch* that resembled, if not wheat; not a *gluten cell found.*

Remarks.—This specimen was really inferior to some common flours.

Twenty two, twenty-three and twenty-four are given in milk, which is the saving element. It should never be forgotten that infants need in food all the chemical elements found in their tissues, and in physiological proportions. Starch and sugar being made up of C. H. O. (three elements), cannot supply the sixteen elements found and needed in the human body. Few, even among physicians, realize the tremendous importance of our subject in a pathological point of view.—*Ephraim Cutter, M.D., in Cin. Med. News.—Sanitary News.*

---

TREATMENT OF HEMORRHOIDS.—Since the time mentioned, I have not used the ligature or ecraseur in a single case, my plan of treatment in confirmed hemorrhoids being very simple, and as follows: all tumors found at the verge of the anus, and covered in part or wholly with integument, are clipped off with the sissors. If situated within the external sphincter, the bowels having been moved with a dose of sulphate of magnesia given a few hours before, the patient is placed over a vessel, and directed to strain (a vessel filled with hot water is best). If the tumors do not come within reach in this way, the finger should be thrust into the bowel, provoking tenesmus, and the patient again instructed to force the piles down. When within reach, the nates being separated by an assistant, the tumors are seized one by one with a forceps, and held, while with the hypodermic syringe, from five to ten minims of a solution of nitrate of silver, one drachm to the ounce of distilled water, are injected into each, not stopping till all have been thus injected. No pain is felt except what is caused by handling parts rendered hyper-sensitive by protracted irritation.—*S. S. Todd, M.D., in St. Louis Courier of Medicine.*

A NEW VERMIFUGE.—In the discussion which followed the narration of these cases, Dr. Harrison Allen said, "I remember seeing in the Patent Office a model of an apparatus for trapping lumbricoid worms. It consisted of a tube fashioned somewhat like a Planten gelatine capsule. An opening was seen on the side, which was continuous with a perforation extending through the short diameter of the tube. A concealed spring occupied one end of the interior and was armed with a number of sharp teeth. The outside was provided with a ring. A long string was tied to this ring. I inferred that the instrument was used in the following manner: The spring being set the apparatus was swallowed by the patient, the string hanging from the mouth. The worm, having thrust a portion of his body through the opening, caused the spring to recoil and the teeth of the trap to pierce the worm and entrap him; the string was then to be used in withdrawing the trap."—*Indiana Med. Journal, September*, 1881.

We knew that application for patents is often made for all manner of conceivable and inconceivable things; but this joke surpasses all. We have known men to fish with worms, but never for worms. This is equal to the well known method of coaxing a coin from the bowels of a man who had swallowed it.

---

A RARE CASE OF SELF-MUTILATION.—Dr. Thiersch related the case of a man who had circumcised himself at the age of eighteen. In 1870, being then married and a father, he slit up the hypogastrium from the symphysis pubis to the umbilicus, so that the omentum protruded; his object being, as he said, to obtain a view of his interior. Although the knife which he used was dirty and blunt, the wound healed after the removal of the omentum. A year later, he laid open one side of the scrotum. The prolapsed testicle was replaced while he was in hospital, and the wound healed without interruption. In 1880, he again laid open his abdomen; the wound healed in fourteen days, notwithstanding prolapse of the omentum. In May, 1880, he removed his right testicle, and himself sewed up the wound. Four weeks later, he treated the left testicle in the same way; the spermatic cord, however, escaped, and a hæmatoma, as large as a child's head, was formed, on account of which he was admitted into hospital. The man acted under the influence of an uncontrolable impulse, and had no quiet until he was completely castrated.—*Med. Gazette, October*, 1881.

---

Cannabis Indica in Migraine.—What the bromides and belladonna are to epilepsy, cannabis indica is to migraine. The principle of treatment laid down is to maintain, by the use of small doses of the agent, a constant influence upon the nervous system for a long time, the same as is required in epilepsy by the use of the bromides. At first, as a matter of course, no appreciable effect is observed, and not until the use of the remedy is persevered in for many weeks, and the nervous system kept under its influence for a considerable time, will the patient find an appreciable diminution in the severity and frequency of the attacks. It is well to commence with one fourth grain of the extract, before each meal, for the first fortnight. The dose may be increased to the third of a grain for the second fortnight, to be augmented to a half grain at the end of four weeks. This amount will generally be sufficient and should be faithfully continued for several months. Success here is only obtained by persevering effort.—*Chicago Med. Jour. and Exam.—Ohio Med. Journal.*

---

Chronic Pelvic Abscess.—In view of these difficulties in diagnosis, which have been acknowledged by the best men in the profession as liable to occur to them, I think it advisable to use the aspirator in all cases of doubtful abdominal tumor before pronouncing definitely upon its nature. This would have prevented most of the mistakes made in the preceding cases.—[Quoted from various reporters.]

In all cases of pelvic abscess, the cavity, after evacuation of the pus, should be kept constantly drained by a syphon drain, and daily washed out with an antiseptic solution. By means of the syphon drain the entrance of air is prevented from without, and hence decomposition of the pus and septicæmia cannot take place. Obliteration of the cavity is also secured more rapidly in consequence of the constant and complete evacuation of its contents.—*A. F. Erich, M.D., in Maryland Med. Journal.*

---

REPORT ON SANITARY SCIENCE.—There is a fatalistic notion which has taken root in the minds of parents, that there are certain classes of affections known as "children's diseases," which all must suffer unless they have a natural immunity. This was the feeling in the eighteenth century in regard to small pox, and then, as now the effort was not so much to avoid infection as to select a favorable season. This is a false philosophy, which must be rooted out of the minds of the people. Let them understand that there are no favorable times for sickness, and then, and not until then will scarlet fever and diseases of its class, cease to send their hecatombs to death every year. Under the same head we may place diphtheria, whoses causes are yet but little known. One thing we know, it is contagious, and the kiss of affection upon the lips of the infected one, has planted the seed of death in the loving parent or child.—*D. N. Kinsman, M.D., in Ohio Med. Journal.*

---

DISSECTION.—The St. Louis *Globe-Democrat* thus has its say about Maine: In Maine they have a law that no medical student shall be allowed to graduate and practice medicine who has not had regular practice in the dissecting room. Then they passed a law that no bodies, save only the bodies of executed criminals, should be cut up in dissecting rooms. Then, as a climax to all this, they abolish capital punishment. That's the kind of a country Maine is. This is like the County Commissioners who passed the following resolutions:

1. *Resolved*, That we build a new jail.

2. *Resolved*, That we build the new jail out of the materials in the old jail.

3. *Resolved*, That we use the old jail until the new jail is finished.—*Med. and Surg. Reporter, September*, 1881.

---

Comparative Mortality.—The six healthiest cities in the United States, as measured by the above and most recent aùthentic reports, up to the time of going to press with this number, were, in the order named:

Utica, Dayton, New Haven, Portland, San Francisco, and Lawrence.

The six unhealthiest were:

Charleston, Memphis, Cleveland, Chicago, Hudson Co., N. J. and Lynn.

The six unhealthiest in the world were:

St. Petersburg, Charleston, Malaga, Alexandria, Wassaw and Budha-Pesth.—*Sanitarian, October*, 1881.

---

Recent Progress in the Treatment of Children's Diseases.—Sometimes when cow's milk cannot be digested ass's or goat's milk is more successful, and sometimes a child, much reduced by digestive disturbance dependent upon an unsuitable dietary, at once recovers when put again to the breast. More often, however, milk of any kind seems to act as an irritant poison, and no hope of relief can be entertained until it is excluded from the diet.—*D. H. Hayden, M.D., in Boston Med. and Surg. Journal.*

An irritant poison, indeed! Verily, one might reasonably infer, from the light shed by some latter day medical writers, that the Almighty had committed a fatal blunder in providing milk, and milk alone, for the nourishment of young children. How, we are led to inquire, did mother Eve raise her boys, in the absence of patent "infants' food" and without the marvelously metamorphosed farinaceous preparations of modern pharmaceutical science—so-called.

---

Medical Societies.—Dr. Robert Barnes says: "The true function of a medical society is to gather together and then diffuse knowledge, to encourage independent inquiry, to survey from time to time, by the light of mutual reflection, the position attained, and thus to seek sound guidance in the application of our knowledge to our practical duties."—*Medical Gazette.*

